# Radial HR-pQCT and Finite Element Analysis in HPP Patients are Superior in Identifying Susceptibility to Fracture-Associated Skeletal Affections Compared to DXA and Laboratory Tests

**DOI:** 10.1007/s00223-023-01082-3

**Published:** 2023-05-06

**Authors:** Felix N. Schmidt, Constantin Schmidt, Julian Delsmann, Michael Amling, Florian Barvencik

**Affiliations:** Department of Osteology and Biomechanics, University Medical Center Hamburg-Eppendorf, Lottestrasse 59, 22529 Hamburg, Germany

**Keywords:** Hypophosphatasia—HPP, Fracture, HR-pQCT, Finite element analysis—FEA, Fracture risk

## Abstract

Hypophosphatasia (HPP) is an inborn disease that causes a rare form of osteomalacia, a mineralization disorder affecting mineralized tissues. Identification of patients at high risk for fractures or other skeletal manifestations (such as insufficiency fractures or excessive bone marrow edema) by bone densitometry and laboratory tests remains clinically challenging. Therefore, we examined two cohorts of patients with variants in the *ALPL* gene grouped by bone manifestations. These groups were compared by means of bone microarchitecture using high-resolution peripheral quantitative computed tomography (HR-pQCT) and simulated mechanical performance utilizing finite element analysis (FEA). Whereas the incidence of skeletal manifestations among the patients could not be determined by dual energy X-ray absorptiometry (DXA) or laboratory assessment, HR-pQCT evaluation showed a distinct pattern of HPP patients with such manifestations. Specifically, these patients had a pronounced loss of trabecular bone mineral density, increased trabecular spacing, and decreased ultimate force at the distal radius. Interestingly, the derived results indicate that the non-weight-bearing radius is superior to the weight-bearing tibia in identifying deteriorated skeletal patterns. Overall, the assessment by HR-pQCT appears to be of high clinical relevance due to the improved identification of HPP patients with an increased risk for fractures or other skeletal manifestations, especially at the distal radius.

**Supplementary Information:**

The online version contains supplementary material available at 10.1007/s00223-023-01082-3.

## Introduction

Hypophosphatasia (HPP) is a hereditary disease that is caused by dysfunction of tissue-nonspecific alkaline phosphatase (TNSALP) caused by mutations in its encoding gene *ALPL* [1]. Five to six different clinical forms, depending on the age at diagnosis, have been described [2], and recently, a genetic-based classification has been proposed [3]. To date, several hundred mutations in the *ALPL* gene have been identified, and they are of particular significance for the development and heterogeneity of the disease. Despite differences in phenotype, HPP is commonly described as a rare form of osteomalacia [4] which arises due to reduced TNSALP activity and results in impaired mineralization of bones and teeth.

**Fig. 1:**
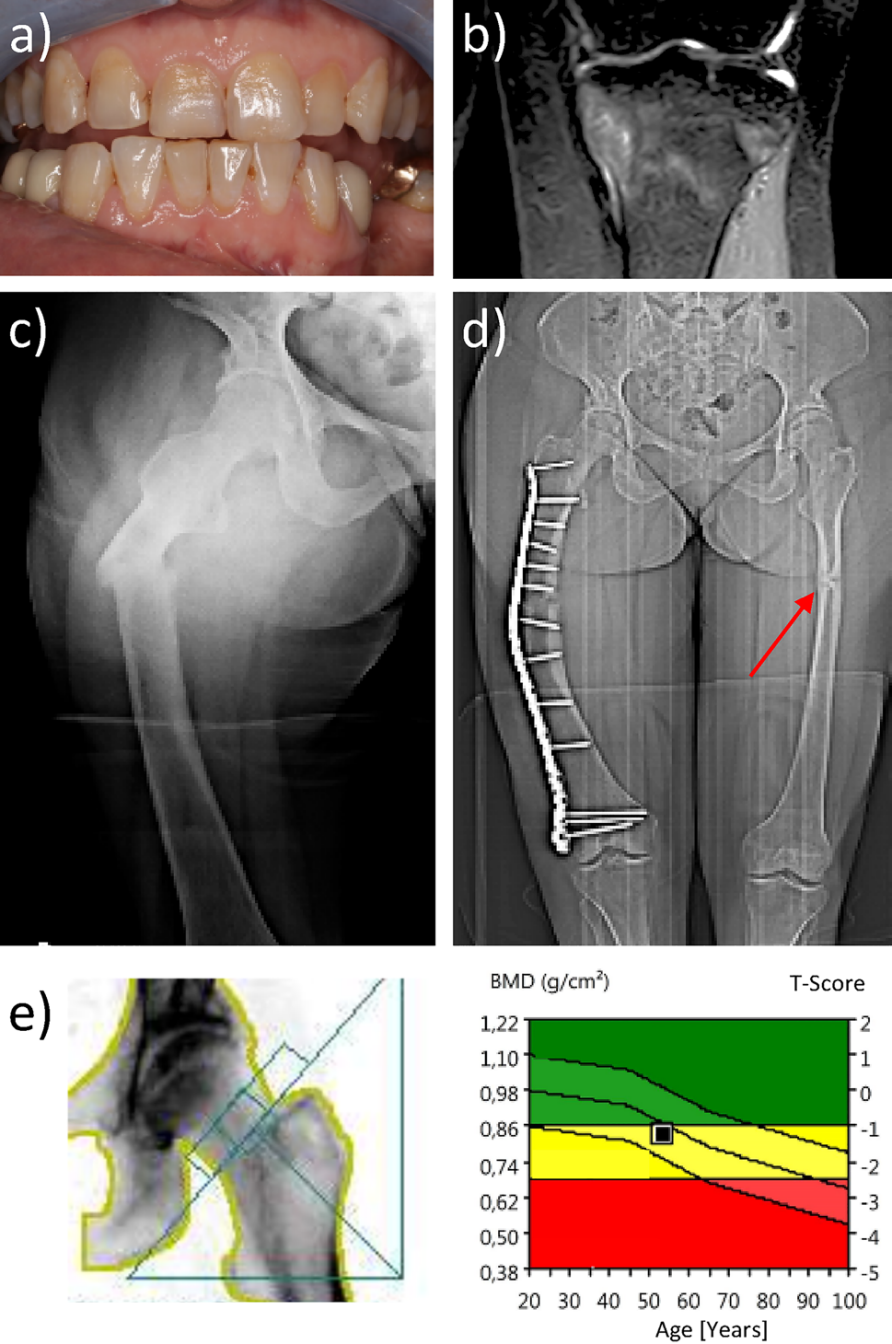
The spectrum of dental and skeletal manifestations of HPP in mineralized tissues: Impaired tooth quality due to hampered cementum mineralization (**a**) is the most common dental sign, while skeletal signs may include bone marrow edema (**b**), low-energy or insufficiency fractures (**c**), pseudofractures (**d**), and nonunions. Despite its frequent use for assessing aBMD (areal bone mineral density) measures obtained by DXA (dual energy X-ray absorptiometry) (**e**) are often within the normal to osteopenic range, suggesting that DXA may have limited value in evaluating the risk of HPP

At the enzymatic level, TNSALP is responsible for making inorganic phosphate available for bone mineralization. Here, TNSALP cleaves pyrophosphate (PP_i_) into inorganic phosphate (P_i_), which is needed for bone mineralization [1], whereas PP_i_ is an effective inhibitor of tissue mineralization [5]. Decreased TNSALP function and thus the accumulation of PP_i_ may lead to extensive mineralization defects in HPP, becoming clinically apparent as insufficiency fractures, reduced mineralization of tooth cementum (Fig. 1a), bone marrow edema (Fig. 1b), fractures (Fig. 1c, d) or nonunions [6].

TNSALP enzyme dysfunction resulting from a genetic defect can cause a wide variety of symptoms [6, 7]. These include symptoms such as tooth loss, joint and bone pain, muscle pain, muscle weakness, migraine-like headaches and gastrointestinal complaints [6, 8, 9]. Furthermore, if fractures occur in HPP, the risk for delayed and impaired fracture healing is increased [10], leading to elevated pain levels, disability and decreased quality of life [11]. Beyond the HPP-related symptoms, patients may have other conditions that are associated with slightly reduced bone quality. Together with HPP symptoms, these can lead to significantly reduced bone quality and make patients susceptible to fractures by structural or compositional deteriorations. Although none of the other conditions alone results in a fatal clinical manifestation, the combination of HPP with one or more of these conditions may hamper bone quality and increase susceptibility to fractures significantly. Therefore, it is important to improve the identification of HPP patients with an elevated risk for skeletal manifestations such as fractures, insufficiency fractures, and bone marrow edema to optimize treatment strategies and enhance preventive methods. This is particularly important given that the onset of HPP in adults does not necessarily indicate the severity of clinical symptoms [12]. If necessary, medications may and can be adapted accordingly.

TNSALP as a widely used analyte in routine clinical practice which can raise suspicion of HPP when activity is low. Most other common bone laboratory values do not indicate changes in laboratory tests in HPP [13, 14], but specific laboratory markers, such as pyridoxal-5-phosphate (PLP), phosphate [8] and phosphoethanolamine (PEA) [8], have been shown to be elevated in fracture patients [8, 9].

Standard diagnostic approaches such as physical examination, X-rays and bone densitometry contribute to fracture risk assessment. Interestingly, T-scores in the spine have been shown to be elevated in patients with fractures [8] compared to those without fractures, but not in the hip (Fig. 1e) [8]. According to Genest et al., this effect may be caused by compensatory increased bone formation; however, three-dimensional image analysis is missing to verify this assumption. An alternative hypothesis suggests a accumulation of phosphate not being incorporated into the bone due to low-performing alkaline phosphatase. Furthermore, lumbar spine T-scores are highly susceptible to structural deteriorations [15] and fractures, a common problem in HPP [16].

**Fig. 2:**
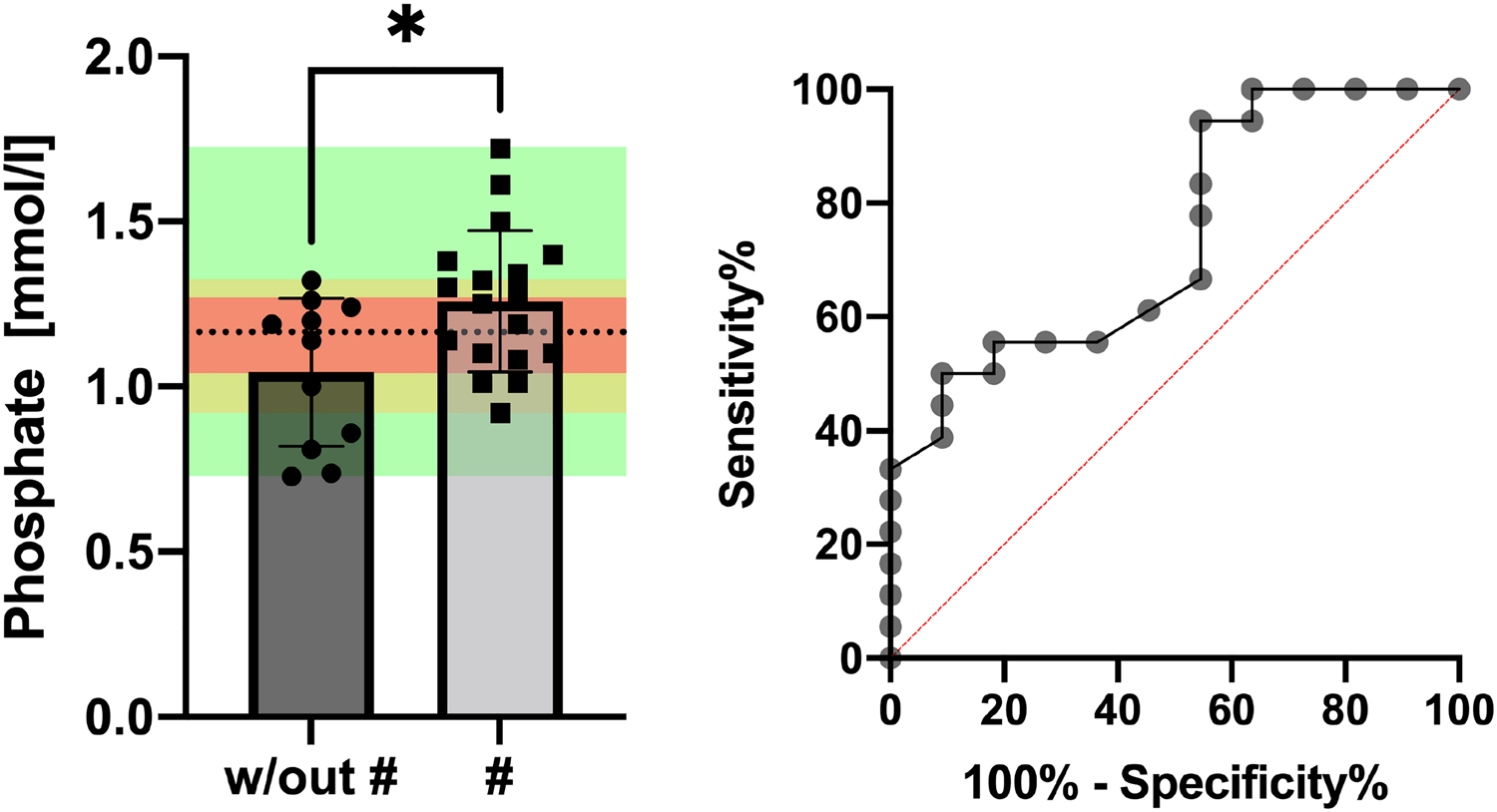
Intersection of the ranges of measured values: Depicted are the phosphate levels of two groups with their indicated means and standard deviations (SDs) significantly differing from each other (*p < 0.05). Based on the intersecting SD of phosphate levels of fractured vs. nonfractured patients (red), 23% of the value range lies within the intersecting standard deviations without group-specific allocation (red). The full range of datapoint intersections is indicated by yellow and includes the majority of datapoints. Data points within the green region are reliably allocable to the specific group (fracture or no fracture) according to the presented dataset but are the minority of values. This visualization clearly demonstrates the need for a parameter with a small intersecting range (red/yellow) to estimate the risk for fracture based on a specific value or to establish a value that can separate the two groups with respect to a threshold where most of the values of one group are below or above and vice versa, as indicated by the dotted line. In the demonstrated case, unfortunately, a large portion of values does not respect the threshold. (B) ROC curve for phosphate clearly shows problems distinguishing between the two groups, visualized by an area under the curve close to 0.5 (angle bisector); w/out #: without fracture, #: fracture

Importantly, given the very high heterogeneity of HPP patients with respect to, for instance, their lumbar spine T-score and phosphate levels [8], the overlap of the value range of fractured and nonfractured patients is at a very high level of close to 50% of the individuals at lumbar spine T-scores (based on the standard deviations [8]). Similarly, the overlap for phosphate serum levels (as indicated in Fig. 2) is approximately 23% with respect to the standard deviations of both groups. This in turn reflects the problem of DXA to reliably detect a fracture-specific pattern due to the overlap of a large portion of the cases, degenerative mechanisms and possible compensatory mechanisms.

There is initial evidence suggesting the inclusion of three-dimensional methods such as HR-pQCT in combination with PLP laboratory tests to identify HPP patients with high fracture risk [9]. However, these results neither exhibit a clear separation of the two groups nor suggest a distinct morpho- or densitometric pattern. Innovative diagnostic tools such as HR-pQCT and finite element analysis (FEA) [17] have the potential to provide a more precise fracture risk prediction in rare diseases like HPP while using very low radiation doses [18].

In HR-pQCT, structural differences in mechanically loaded (tibia) and unloaded (radius) areas can be measured. Here, we examined the bone microstructure, bone mineralization and bones simulated mechanical performance (FEA) of patients with HPP. We hypothesized that HPP patients with fractures have inferior bone microarchitecture, mineralization, and mechanical performance compared to those without fractures. It is important to note that this hypothesis does not address whether the measured effect comes from a more severe form of HPP or whether it is caused by additional propensities of the individual due to other side conditions. However, the hypothesis is that HPP patients prone to fractures can be differentiated by means of bone structure and composition. In addition, we hypothesized that bones with high loading (tibia) would be a less suitable region to study for fracture susceptibility than areas of low loading, such as the radius.

## Materials and Methods

### Study Group

The 45 retrospectively analyzed patients included in this report were seen in our outpatient clinic (Department for Osteology and Biomechanics, University Medical Center Hamburg-Eppendorf, Germany) between 2016 and 2020. All patients were diagnosed with HPP based on clinical examination and genetic analysis, including persistently elevated PLP serum levels (> 27.1 µg/L) as well as clinical symptoms and family history. Therefore, all patients exhibited at least two typical symptoms of HPP and an ACMG (American College of Medical Genetics and Genomics) class III–V [19] variant or clinically pathogen variant (ClinVar) in the *ALPL* gene (cf. Suppl. Table 1). All patients received a standardized clinical interview including medical history, drug treatment, fracture and bone pain history, family history, symptom history and a clinical examination. Other causes of hypophosphatasemia including treatment with bisphosphonates, denosumab or steroids, multiple myeloma, osteogenesis imperfecta, renal dystrophy (Krea > 1,3) and vitamin D overdosing (Vit. D > 100 µg/L) and underweight (body mass index (BMI) < 18.5) were excluded. Two groups were formed based on the medical history and current symptoms, that is, one group comprised all patients without skeletal manifestations (w/out #) in terms of fractures, defined as fractures, insufficiency fractures or bone marrow edema (bone bruise) in the medical history accessed by a clinical interview. Bone deformities were not included in the fracture group and were excluded from the study if clinically apparent. The other group included patients suffering from the aforementioned symptoms (Suppl. Table 1). Patients with bone-affecting medication, cancer, glucocorticoid treatment, and other metabolic bone diseases or severe underweight were excluded. Further risk factors were assessed (Suppl. Table 1). This retrospective study was performed in accordance with the local ethical guidelines and the Declaration of Helsinki. In the group of non-bone-affected patients, the sex ratio was 0.62, and in the group compared, the sex ratio was 0.72 (coding 0 for male and 1 for female) with a p value of *p* = 0.54 by McNemar testing, indicating no differences in sex distribution between the groups (see Table 1).

**Table 1:** Group specifics: Mean values are presented with the standard deviations (SDs) for the bone affected (#) and nonaffected group (w/out #)

	w/out # [mean ± SD]	# [mean ± SD]	*p* value
Age (Years)	47.813 ± 11.12	50.11 ± 12.17	0.51
Sex	61.54%	72.22%	0.54
Height (m)	168.34 ± 10.61	166.83 ± 10.63	0.71
Weight (kg)	71.46 ± 12.79	70.66 ± 20.44	0.91
BMI (cm^2^/kg)	25.20 ± 0.53	25.06 ± 4.88	0.94

*p values are presented to indicate whether the differences were significant. The sex ratio is presented as the percentage of female individuals*

### Biochemical Analysis

On the day of the clinical visit, blood samples were routinely collected from all patients who were instructed to not have supplemented their diets with vitamin B_6_ or calcium within the last 4 weeks prior to blood sampling. Calcium, phosphate, parathyroid hormone (PTH), bTNSALP/bALP and TNSALP/ALP, osteocalcin, 25-OH-D_3_ (25-hydroxyvitamin D) and the urinary levels of deoxypyridinoline/creatinine (DPD/crea) were measured at the Department of Clinical Chemistry, University Medical Center Hamburg-Eppendorf. Pyridoxal-5-phosphate (PLP) was measured using high-performance liquid chromatography following derivatization with fluorometric detection.

### DXA

In all patients included, areal bone mineral density (aBMD) measurement by dual energy X-ray absorptiometry (DXA) (Lunar iDXA, GE, Madison, WI, USA) was available for analysis. The left and right proximal femur as well as the lumbar spine (L_1_–L_4_) were scanned and evaluated according to the manufacturer’s manual within the clinical routine protocols in accordance to the ISCD [20]. From the measured areal bone mineral denisty (aBMD), the respective T-scores were calculated. For femoral T-scores, the lowest parameter of the left and right site per individual was chosen, choosing from total proximal femur and the femoral neck measurement. For the lumbar spine, the average value of L_1_–L_4_ was used.

### HR-pQCT

Forty-five patients were measured using a first-generation HR-pQCT (XtremeCT, Scanco Medical AG, Brüttisellen, Switzerland). The settings were set to the manufacturer’s standard clinical settings (60 kVp, 1000 μA, 100 ms integration time and voxel size of 82 μm), and the reference was placed according to [18] with a fixed proximal offset from the reference line. The nondominant radius and contralateral tibia were scanned, if possible. In the case of metal implants and fractures within the last 10 years in the area of HR-pQCT scans, the measurement was performed on the unaffected site. All scans were visually checked for motion artifacts according to the manufacturer and Pialat et al. [21, 22]. Sixteen patients exhibited motion artifacts beyond grade 3 in one of the HR-pQCT scan sites and were excluded from the evaluation. Trabecular area (Tb.Ar), cortical area (Ct.Ar), trabecular and cortical bone mineral density (Tb.BMD, Ct.BMD) as well as total area density (Tt.BMD) were calculated as well as structural parameters cortical thickness (Ct.Th), trabecular thickness (Tb.Th), trabecular spacing and number (Tb.Sp and Tb.N). The aforementioned parameters were subsequently calculated in terms of Burt et al. [23]. Additionally, the manufacturer’s finite element protocol was carried out on the datasets, and stiffness, ultimate force (F_ult_), apparent modulus and fraction of force loaded to the proximal and distal cortex were calculated. For Young’s modulus, 10 GPa was set. F_ult_ was subsequently expressed as a percentage of the age-matched reference group according to Burt et al. [23] prior to further analysis. Trabecular and cortical *von Mises* stresses (Tb.vMS and Ct.vMS) were calculated with the manufacturer’s protocol with respect to the BMD of the respective patient measurement.

### Statistics

Statistical analysis was carried out comparing a group of patients without skeletal manifestations (as mentioned above; referred to as w/out #) and patients with manifestations (referred to as #). Each parameter was tested for normality using the Kolmogorov‒Smirnov test. In the case of normal distribution, a two-sided, unpaired Student’s *t* test was used. In the case of a nonnormal distribution, the Mann‒Whitney test was used. *P* values below 0.05 were considered to indicate significant differences. ROC (receiver operating characteristic) analysis was carried out with p values below 0.05 indicating significance and a high area under the curve to go along with good separation and prediction of the group classification. Additionally, the Tb.N and Tb.BMD of the distal radius was multiplied and tested for significant differences and subjected to ROC analysis. For statistical analysis, SPSS 25 (IBM, Armonk, United States of America) was used, and graphs were drawn using GraphPad PRISM 9 (GraphPad Software, Inc., United States of America). For calculation of the full SD interval, the respective SD was added to the higher mean, and the corresponding SD of the lower mean was subtracted. The subsequently facing tails of the SD were added to the corresponding mean value, and the interval was calculated by subtraction and taking the absolute of the result. Therefore, the percentual intersection of the two means and standard deviations was calculated as the portion of the full SD interval calculated in the first step and stated as the intersection [%]. Parameters with an intersection of less than 10% were considered especially valuable by their good separation, expressed as a low overlap of the value distributions of the two groups.

## Results

### Study Group

**Fig. 3:**
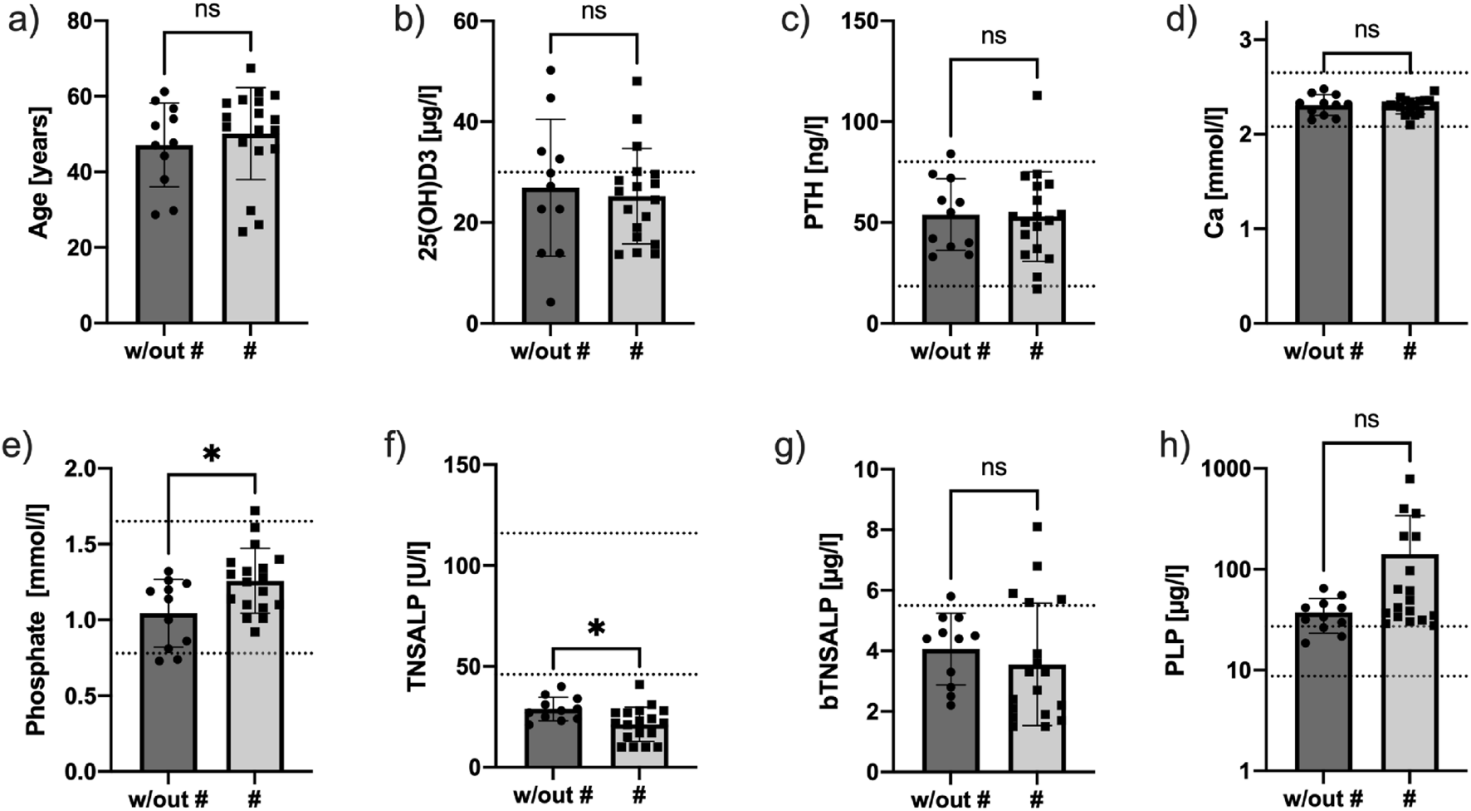
Laboratory evaluation of study subjects: The groups without (w/out #) and with (#) bone manifestation were not significantly different in terms of age (**a**), vitamin D (**b**), parathyroid hormone (PTH) (**c**) or calcium (Ca) (**d**) serum levels. Phosphate serum levels were higher in the group with bone manifestations (**e**). TNSALP/ALP (**f**) levels were significantly lower in the # group; however, bTNSALP/bALP (**g**) levels did not differ between both groups, and PLP levels (**h**) tended toward higher values in the # group. Reference ranges are indicated by dotted horizontal lines; *p ≤ 0.05; w/out #: without fracture, #: fracture, ns: not significant, 25(OH)D3: 25-hydroxyvitamin D, *PTH*: parathyroid hormone, *Ca*: Calcium, *TNSALP:* tissue-nonspecific alkaline phosphatase, *bTNSALP*: bone tissue-nonspecific alkaline phosphatase, *PLP*: pyridoxal-5-phosphate

Patient age did not significantly differ between the groups (47.13 ± 11.12 vs. 50.11 ± 12.17 years, *p* = 0.515, Fig. 3a).

### Biochemical Analysis

Whereas both groups had vitamin D levels below 30 µg/L, there were no differences between individuals with and without skeletal manifestations, with a mean level of 26.91 ± 13.57 µg/L vs. 25.25 ± 9.47 µg/L (*p* = 0.707) (Fig. 3b). PTH (parathyroid hormone) levels did not differ significantly between the groups (*p* = 0.909, Fig. 3c) in terms of serum calcium (2.31 ± 0.11 mmol/L vs. 2.399 ± 0.084 mmol/L, *p* = 0.800, Fig. 3d), while phosphate levels were significantly higher in the skeleton-affected group (1.05 ± 0.22 mmol/L vs. 1.26 ± 0.21 mmol/L, *p* = 0.016, Fig. 3e). Tissue-nonspecific alkaline phosphatase (TNSALP/ALP) levels were significantly lower in the group with skeletal manifestations (*p* = 0.014, Fig. 3f), and a similar trend was observed for bTNSALP. Neither osteocalcin as a bone formation marker nor the urine-DPD levels normalized to creatinine differed significantly between the groups (*p* = 0.977 and *p* = 0.903, respectively). PLP levels tended toward higher values in the group with skeletal manifestations (*p* = 0.061, Fig. 3h). A ROC analysis of phosphate levels in relation to their ability to assign association to the bone-affected group or group without bone affection showed the moderate benefit of this serum parameter (area under the curve = 0.7399, *p* = 0.033). A subsequent ROC analysis for PLP levels showed an area under the curve of 0.7121 (*p* =  0.059) for PLP levels.

### DXA Measurements

**Fig. 4:**
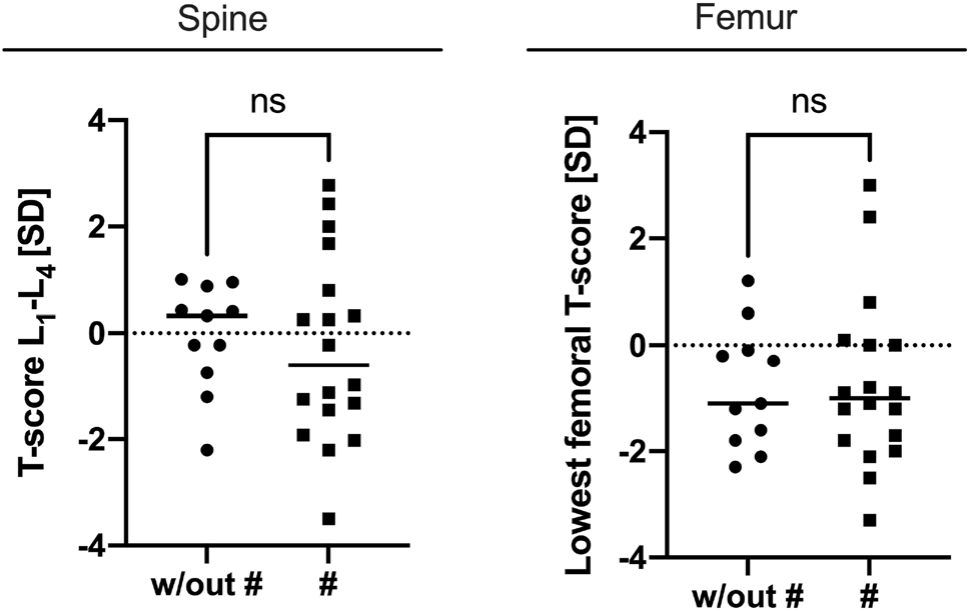
DXA measures of the two groups: No significant differences were found in any of the lumbar vertebrae L_1_–L_4_ (Spine) or in the left or right femur (Femur) in the presented groups of this study; w/out #: without fracture, #: fracture, *ns:* not significant, *SD*: standard deviation

Between the groups, no differences with respect to DXA measurements were detected either in the lumbar spine T-score (L_1_–L_4_, Fig. 4) or in the proximal femur (Fig. 4). The T-score of the lumbar spine was centered close to zero without significant differences (*p* = 0.672, Fig. 4). Differences between the lowest femoral T-scores did not reach significance with a p value of 0.892 unsuitable to differentiate between the groups via DXA. Individuals with a high lumbar spine bone mass with a T-score above 1 did not present with higher HR-pQCT structural values than other individuals of the #-group.

### HR-pQCT:

**Fig. 5:**
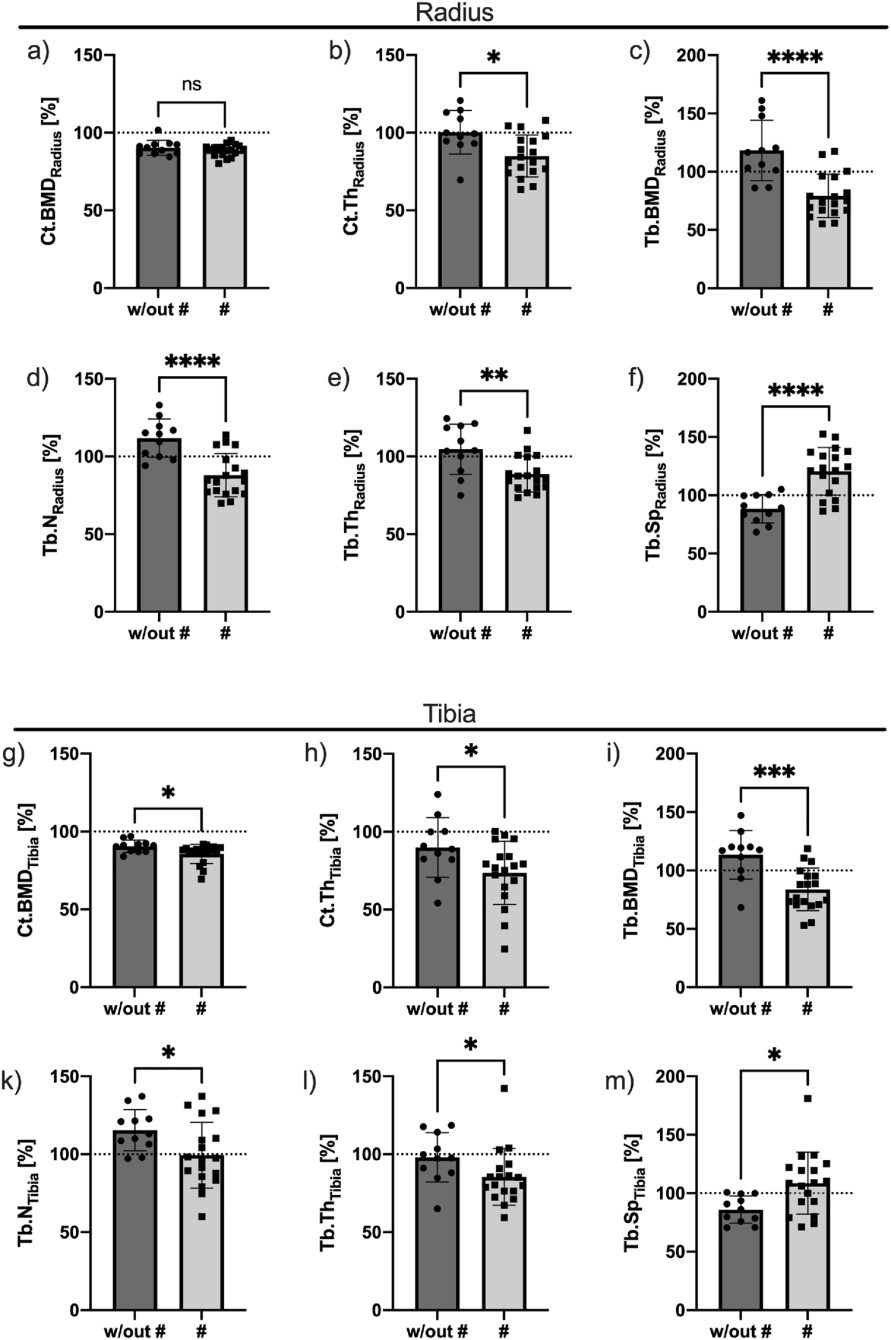
HR-pQCT results in relation to an age- and sex-specific reference group in percent: In the radius, no difference was detected in the cortical BMD; however, both groups had a lower Ct.BMD (< 90%) than the reference (**a**). All other presented density and morphometry parameters of the radius (**b**–**e**) were significantly lower in the # group with a corresponding higher trabecular spacing (**f**). The highest p values with respect to differences between the groups were found for trabecular bone mineral density (Tb.BMD—**c**), trabecular number (Tb.N—**e**) and corresponding trabecular spacing (Tb.Sp—**f**). Notably, for the latter parameters, only max. 4 individuals did not undermatch (Tb.BMD & Tb.N) or overmatch the 100% reference mark with respect to the group classified as fractured, indicating low intersection compared to the same parameters in the tibia. In the tibia, the same pattern of relative differences in the two groups was observed; however, p values indicated weaker differentiation (**g**-**m**) and a higher intersect of the groups than in the case of the radius. *p ≤ 0.05, **p ≤ 0.01, ***p ≤ 0.005, ****p ≤ 0.001; w/out #: without fracture, #: fracture, *ns:* not significant, *Ct.BMD:* cortical bone mineral density, *Tb.BMD*: trabecular bone mineral density, *Ct.Th:* cortical thickness, *Tb.N*: trabecular number, *Tb.Th*: trabecular thickness, *Tb.Sp:* trabecular separation

At the distal radius, mean Ct.BMD was measured at approximately 90% of the reference group in both groups, and there was no significant difference (Fig. 5a). Ct.Ar was significantly lower in the bone-affected group than in the nonaffected group, with a mean reduction of 16.90% (w/out #: 112.10% ± 13.57% vs. #: 93.15% ± 15.70%, *p* ≤ 0.005). In contrast, Tb.Ar did not differ between the groups (w/out #: 85.75% ± 19.33% vs. #: 86.52% ± 15.53%, *p* = 0.906). The results of the HR-pQCT analysis are summarized in Table 2.

**Table 2:** HR-pQCT parameters and FEA-derived F_ult_: HR-pQCT parameters are displayed as the percentage of the age-matched reference

	w/out # [%]	# [%]	p value	Intersect [%]
Radius				
Ct.Ar	112.10 ± 13.57	93.15 ± 15.70	**0.0026**	21.40
Tb.Ar	85.75 ± 19.33	86.52 ± 15.53	0.9058	95.63
Tt.BMD	121.15 ± 16.17	97.83 ± 13.81	**0.0003**	12.49
Ct.BMD	89.15 ± 4.86	88.05 ± 4.42	0.2821	65.92
Tb.BMD	118.34 ± 25.94	79.19 ± 18.60	** ≤ 0.0001**	**6.43**
Ct.Th	100.32 ± 13.99	84.95 ± 13.47	**0.0067**	28.25
Tb.N	111.72 ± 12.32	87.98 ± 13.91	** ≤ 0.0001**	**5.00**
Tb.Th	104.63 ± 16.17	88.70 ± 11.63	**0.0046**	27.15
Tb.Sp	88.47 ± 12.17	120.54 ± 20.51	** ≤ 0.0001**	**0.93**
F_ult_	200.23 ± 36.21	156.87 ± 30.25	**0.0017**	21.05
Tibia				
Ct.Ar	104.71 ± 24.49	80.62 ± 21.31	**0.0110**	32.08
Tb.Ar	107.33 ± 25.03	98.50 ± 25.44	0.3694	70.21
Tt.BMD	105.84 ± 16.17	86.20 ± 16.79	**0.0045**	25.34
Ct.BMD	90.47 ± 3.95	85.60 ± 6.12	0.0266	34.87
Tb.BMD	113.38 ± 20.80	83.82 ± 18.31	**0.0004**	13.92
Ct.Th	89.85 ± 19.10	73.55 ± 20.26	**0.0408**	41.39
Tb.N	115.46 ± 13.23	99.43 ± 21.09	**0.0324**	36.32
Tb.Th	98.00 ± 15.83	85.49 ± 18.19	0.0703	46.22
Tb.Sp	85.99 ± 11.58	108.63 ± 26.49	**0.0127**	25.41
F_ult_	204.19 ± 43.82	147.69 ± 34.51	**0.0007**	16.43

Additionally, intersect [%] indicates the percentage of intersection of the two groups’ value range with respect to one standard deviation referring to the age- and sex-specific 100% line. The smaller the intersect, the better the separation. Intersections below 10% are indicated in bold

*Ct.Ar:* cortical area, *Tb.Ar*: trabecular area, *Tt.BMD*: total bone mineral density, *Ct.BMD*: cortical bone mineral density, *Tb.BMD*: trabecular bone mineral density, *Ct.Th:* cortical thickness, *Tb.N*: trabecular number, *Tb.Th:* trabecular thickness, *Tb.Sp:* trabecular separation, *F*_*ult*_: ultimate force, w/out #: without fracture, #: fracture

Values for Tt.BMD in the radius were 19.25% lower in the group with skeletal manifestations (w/out #: 121.15% ± 16.17% vs. #: 97.83% ± 13,81%, *p* = 0.0003). No differences were detected between the Ct.BMD values (*p* = 0.282, Fig. 5a). Ct.Th was significantly lower in the bone-affected group (84.95 ± 13.47) than in the nonaffected group (100.32% ± 13.99), with a p value *p* = 0.007 (Fig. 5b). Tb.BMD was measured to be on average 33.08% lower in the bone-affected group than in the nonaffected group (w/out #: 118.34% ± 25.94% vs. #: 79.19% ± 18.60%, *p* < 0.0001, Fig. 5c). In the group with skeletal manifestations, a lower Tb.Th (w/out #: 104.63% ± 16.17 vs. #: 88.70 ± 11.63, *p* = 0.0046, Fig. 5d) and Tb.N (w/out #: 111.72% ± 12.32 vs. #: 87.98 ± 13.91, *p* ≤ 0.0001, Fig. 5e) and a correspondingly higher Tb.Sp (w/out #: 88.47% ± 12.17 vs. #: 120.54 ± 20.51, *p* ≤ 0.0001, Fig. 5f) were observed. Notably, in Tb.Sp in the radius, only one individual in the bone nonaffected group exceeded 101% of the reference value of the matched reference group (Fig. 5f), and only four individuals fell below the 100% mark in the affected group (Fig. 5f). A ROC analysis was carried out on Tb.N, Tb.Sp, Tb.BMD, (Fig. 7a–c) and Tt.BMD in the radius. The results are summarized in Table 3. Tb.N, Tb.Sp and Tb.BMD revealed the highest areas under the curve quantifying the ability to discriminate between individuals with bone affection and individuals without. ANCOVA (analysis of covariance) for radial Tb.N, Tb.Sp and Tb.BMD was additionally calculated, including age as a covariate. For all parameters, age was not significant.

**Table 3:** Summary of the ROC analysis of HR-pQCT parameters derived from structural analysis, density quantification and FEM

	Area (C-index)	p value	Percent of reference	sensitivity	specificity
Radius					
Tb.N	0.9040	< 0.0005	< 101.1	77.78	72.73
Tb.Sp	0.8939	0.0005	> 103.6	72.22	90.91
Tb.BMD	0.9141	< 0.0005	< 100.8	88.89	81.82
Tt.BMD	0.8636	0.0012	< 106.4	83.78	90.91
F_ult_	0.8647	< 0.0005	< 183.7	84.21	71.43
Tibia					
Tb.N	0.7374	0.0346	< 109.6	72.22	63.64
Tb.Sp	0.7980	0.0080	> 91.63	77.78	63.64
Tb.BMD	0.8586	0.0014	< 111.0	94.44	72.73
Tt.BMD	0.7980	0.0080	< 107.4	94.44	72.73
F_ult_	0.8586	0.0014	< 176.0	83.33	72.73

Only parameters with a p value below 0.009 were analyzed

*Tt.BMD*: total bone mineral density, *Tb.BMD*: trabecular bone mineral density, *Tb.N*: trabecular number, *Tb.Sp:* trabecular separation, *F*_*ult*_: ultimate force

At the distal tibia, the mean Ct.BMD of both groups was clearly below 90% of the respective reference group (Fig. 5g). Ct.Ar was significantly lower in the bone-affected group than in the nonaffected group, with a mean reduction of 23.01% (w/out #: 104.71% ± 24.49% vs. #: 80.62% ± 21.31%, *p* = 0.011). Tb.Ar did not differ between the groups (w/out #: 107.33% ± 25.03% vs. #: 98.50% ± 25.44%, *p* = 0.369). Individuals without skeletal manifestations had significantly higher parameters of Tt.BMD and Ct.BMD (Fig. 5g). Tb.BMD (Fig. 5i) did differ between the two groups, as presented in Table 2. Cortical thickness was lower in the affected group, as were Tb.N and Tb.Th and a higher Tb.Sp was present (Fig. 5k–m).

### HR-pQCT FEA

**Fig. 6:**
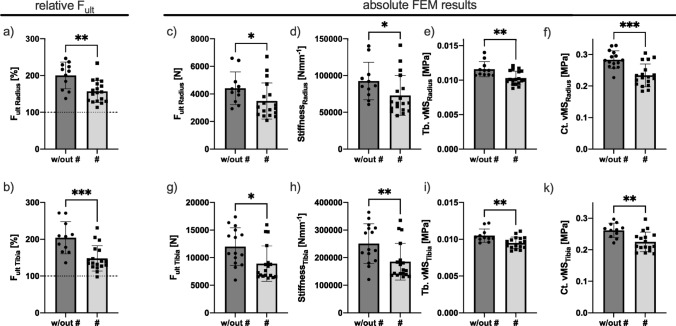
Finite element analysis of the two groups: In the comparison of the relative FEA results with respect to age- and sex-matched reference values, patients with bone manifestations did exhibit inferior mechanical performance represented by a lower ultimate force (F_ult_) in the radius (**a**) and tibia (**b**). Relative values are higher than the mated reference due to different assigned Young’s moduli for FEA by Burt et al. Absolute values **c**–**k** did present the affected group to have inferior mechanical performance. Notably, as in structure and mineralization, differences were more pronounced in the radius (**c**–**f**) than in the tibia (**g**–**k**). Our results indicate that the trabecular (**e, i**) and cortical (**f, k**) compartments have an inferior structure with lower *von Mises* stresses (vMS) in the affected group. *p ≤ 0.05, **p ≤ 0.01, ***p ≤ 0.005; w/out #: without fracture, *F*_*ult*_: ultimate force, #:  fracture; *Ct.vMS*: cortical *von Mises* stresses, *Tb.vMS:* trabecular *von Mises* stresses

FE simulation was evaluated on the radius and tibia and did exhibit significantly lower F_ult_ values with respect to the matched reference group in the bone-affected individuals at the distal radius and tibia (Table 2, Fig. 6a, b). Direct, nonnormalized results are presented for F_ult_ and stiffness (S), with significantly lower values in individuals with skeletal manifestations (Fig. 6c, d, g, h). With respect to the BMD depended FEA, *von Mises* stresses in trabecular bone and cortical bone (Tb.vMS and Ct.vMS) in the radius and tibia had significantly lower values in the affected group for the radius and tibia with lower p values in the radius (Fig. 6e, f, i, k). Four individuals had higher values, which were linked mainly to Tb.N and Tb.BMD but were not linked to laboratory values or type of manifestation.

### ROC Analysis

**Fig. 7:**
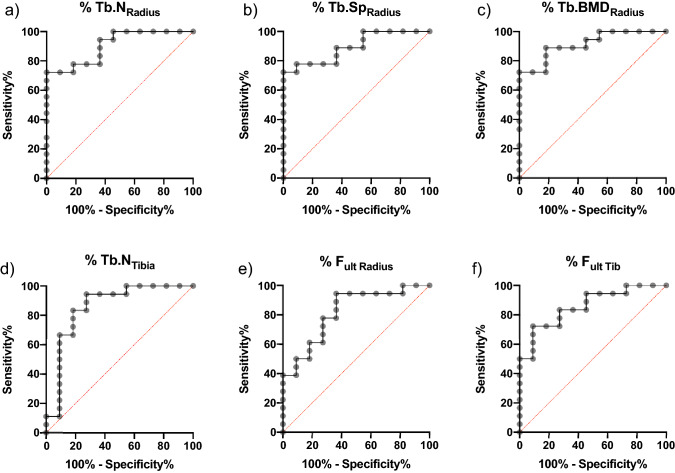
ROC analysis of specific parameters with low intersection: The higher and more rectangular the curve is, the better the prediction of the parameter. Tb.N (**a**) and Tb.Sp (**b**) in the radius and Tb.BMD of the radius (**c**) had areas under the curve > 0.85. Tb.BMD of the radius (**c**) was superior to Tb.BMD of the tibia (**d**). Interestingly, FEA analysis of the radius was inferior using relative F_ult_ (**e**) compared with the F_ult_ of the tibia (**f**), with a slightly higher area under the curve. Interestingly, FEA values (**e**–**f**) were less sensitive at high specificity than structural parameters Tb.N and Tb.Sp (**a**, **b**). *Tb.BMD:* trabecular bone mineral density, *Tb.N*: trabecular number, *Tb.Sp*: trabecular separation, *F*_*ult*_: ultimate force

ROC analysis was carried out on parameters that significantly differed with a p value *p* < 0.009. The highest areas under the curve as a measure of sensitivity and specificity were Tb.N_Radius_ and Tb.Sp_Radius_ (Fig. 7a, b) as well as Tb.BMD_Radius_ (Fig. 7c) with areas under the curve (AUCs) of 0.9040, 0.8939 and 0.9141, respectively. This means for a parameter such as Tb.N in the radius, 90.40% of the cases with fracture will have a lower measure than a nonaffected patient (Table 3). Additional parameters with high AUC were Tb.BMD of the tibia (Fig. 7d) and ultimate force of the radius and tibia (Fig. 7e, f). Characteristic values of the most different parameters are presented in Table 3.

Multiplication of the two parameters with the highest AUC, namely, Tb.BMD and Tb.N of the radius, revealed an area under the curve of 0.9192 and a p-value of 0.0002. ROC analysis of Tb.vMS and Ct.vMS showed a significant differentiation with higher AUCs in the radial group than in the tibial group for both parameters.

## Discussion

Enabling clinicians to link the occurrence of fractures (Fig. 1c) and repetitive fractures (Fig. 1c, d) most reliably to diagnostic parameters is of crucial importance for identification and adequate treatment of HPP patients with high fracture risk.

We compared two groups of patients suffering HPP distinguished solely by the clinical appearance of bone manifestations, namely, fractures, insufficiency fractures and bone edema, to determine whether there is a difference in structural or mineralization pattern between these two groups. Therefore, bone-related predispositions, regardless of the HPP, may be present at a subclinical level within the normal distribution of a healthy cohort. However, in addition to the HPP, they may combine to increase susceptibility to fractures. In other words, patients may compensate for the HPP manifestation itself if the remaining predisposing factors favor good bone quality, but an additional tendency toward lower yet normal bone parameters may slightly hamper bone quality. Together with HPP, this may contribute to higher fracture occurrence.

In our study, there was no difference in age or sex between the groups; however, both groups had a vitamin D level below the recommendation of 30 µg/L, with no significant difference. The particularly low vitamin D levels in some individuals of the unaffected group (Fig. 3b) would be expected to enhance susceptibility to fractures, but fractures were not present in this group.

No differences in the severity of the *ALPL* gene mutation were observed between the groups (Suppl. Table 1). Both groups had comparable heterozygous mutations, suggesting that fracture occurrence is not linked to the severity index of mutations or the age of onset [12]. Phosphate levels were elevated in bone-affected individuals compared to nonaffected individuals (Fig. 3e), in line with the literature [8]. Unlike other forms of osteomalacia, HPP patients exhibit normal-to-high levels of phosphate and calcium [13]. P- and PLP levels, known as potential markers [8, 9] for HPP, did indicate a moderate (P) to nonsignificant (PLP) ability to separate the groups with and without bone manifestations in ROC analysis. This indicates a separation of the groups by their mean values, as shown by Schmidt et al. [9]; however, the intersection of the range of values of the parameters was high, motivating the need for additional parameters to further describe and differentiate the two groups. Since HPP is described as a mineralization disorder [1, 14], it becomes evident that mineral quantification methods, such as DXA (Fig. 1e, f), may be of further help.

DXA is the gold standard in clinical bone densitometry, separating patients with and without fractures in osteoporosis [24]. Our results did not show significant differences between HPP patients with and without bone manifestations. However, higher heterogeneity was present in the affected group, in line with the literature [8]. A subset of four patients had high lumbar spine T-score > 1.5 in DXA but did not separate in HR-pQCT imaging. Previous studies have reported higher lumbar spine T-score in fracture subgroups in larger cohorts [8], yet with a large overlap between the groups, corresponding to approximately 50% of individuals. Therefore, our results indicate the challenging task of differentiating affected and nonaffected patients using DXA [9] highlighting the need for innovative, advanced imaging methods to improve the accuracy of group differentiation.

HR-pQCT, an innovative and advanced method, is able to differentiate between structural parameters and bone mineral content, providing a precise measure of bone compartments [18] and enabling virtual mechanical testing [17].

Compared to reference values [23], Ct.BMD is generally decreased in HPP patients (Table 2), in line with previous reports [9]. This effect is also described for other mineralization disorders, such as X-linked hypophosphatemic rickets [25]. A lower cortical thickness was present in fractured cases in line with former HR-pQCT analysis [9] and radiographic results [26]. As the cortical compartment carries a relevant amount of load [27], this difference may be part of the susceptibility of these bones to fractures [28].

The trabecular compartment of the radius showed major, significant differences, which were more pronounced than in the tibia. Our data suggests that the radius is the superior region of interest to detect a specific pattern of HPP patients with a high risk for bone fractures, insufficiency fractures and/or bone marrow edema in HR-pQCT. Especially in the radius, the mineralization of the trabecular network (Tb.BMD) was lower in the affected group, in line with previous reports [9]. Thus, insufficient mineralization provokes the accumulation of fractures or other bone manifestations due to insufficient mechanical load-bearing capacity [29]. Therefore, Tb.BMD_Radius_ can serve as an imaging parameter to estimate the severity of the disease with a low number of overlapping individuals (Fig. 5c). Interestingly, a lower mineral content was not detected via DXA (Fig. 4) or even found to be higher in other studies [8], highlighting the advantages of three-dimensional imaging via HR-pQCT measurement at the periphery. Structurally, Tb.N and Tb.Sp had the best separation between the two groups with overlaps below 10% in the radius. HPP is a lifelong disease [1] with an inherent lack of substrate (P_i_) for bone formation [1]. As a result it causes a thinned-out trabecular network, which is most prominently reflected in a lower Tb.N_Radius_ and higher Tb.Sp_Radius_, especially in the radius, where only a few individuals intersect between the groups (Fig. 5e, f, Table 2).

The radius experiences a lower mechanical stimulus for bone formation than the tibia, which may explain the accentuation. Particularly in a deficit situation of P_i_ for mineralization of osteoid, such as in the case of HPP [26], the higher loads in the tibia may stimulate the remaining TNSALP action. Other osteocyte-driven factors, such as local sclerostin levels, are higher in mechanically unloaded regions, such as the radius [30]. This can favor an inferior bone structure. Thus, the effect of decreased functionality is especially pronounced in the radius with a lower need for mineralization according to Wolff’s law and the mechano-stat model [31].

These observations indicate that HR-pQCT parameters of patients suffering from any kind of fracture are inferior to those of individuals without clinically apparent bone manifestations. Therefore, HR-pQCT is proposed as an important tool to enable clinicians to separate HPP patients with and without a high risk of fractures. However, it is important to note that the observed pattern of reduced bone quality may be partially attributed to factors independent of HPP. Thus, it can be said that HPP patients with fractures clearly present with inferior parameters but are not necessarily caused by HPP. Nevertheless, it must be said that HR-pQCT is a recently emerging and thereby expensive method (compared to DXA) that is not yet widely available and is therefore only available to specialized centers today. Nonetheless, and especially for these centers, as well as an increasing use of HR-pQCT in the future, a profound understanding is of utmost importance.

Finite element analysis (FEA) is known to be a good predictor of fractures [32] and enables clinicians to perform a virtual mechanical bone compression test [27]. In our study, FEA results for radius and tibia did significantly separate patients with fractures and without. However, separation was less pronounced with FEA than with mineralization or density parameters. Of note, the calculated relative F_ult_ values were considerably higher than those in the reference group [23], which can be explained by the different FEA methods and higher (10 GPa) modulus in our simulation. ROC analysis clearly indicate that Tb.N_Radius_ and Tb.Sp_Radius_, as well as Tb.BMD_Radius_, are the features that best separate fracture appearance, also in comparison to FEA parameters.

Interestingly, these patterns seem specific to HPP since other parameters have been shown to be most specific for osteoporosis-related fractures [33], yet the general favorability of the radius is consistent [32, 34]. It should be emphasized that Tb.Sp and Tb.BMD in particular are directly calculated parameters of HR-pQCT and are therefore quite reliable. Additionally, the measured changes meet the criteria reported by Mikolajewicz et al. [33] with respect to least significant changes between the two groups, once again indicating the high contrast ability of these parameters.

Despite its novelty, this study has several limitations. Regarding the study design, we have not been able to prospectively check if an occurrence of a fracture is linked to HR-pQCT parameters. Therefore, future studies are needed to check the validity of the parameters as good predictors. Furthermore, future studies are needed to unravel the cause of differing structure and mineralization features and their interaction with laboratory tests. However, correlation analysis of the PLP or TNSALP levels with Tb.Sp did not reveal a significant association, highlighting the need for explanations beyond the biochemical activity of TNSALP, which again highlights the clinical relevance of HR-pQCT for detecting patients with fractures by focusing on named parameters.

## Conclusion

Taken together, our results clearly indicate that DXA is not capable of separating patients with fracture or bone marrow edema from those without these conditions by means of areal bone density evaluation in the lumbar spine or hip. HR-pQCT has a superior ability to separate HPP patients with and without fractures concentrating on the radial trabecular mineral content, number and separation. Therefore, it can be concluded that HR-pQCT is a relevant diagnostic tool in the stratification of HPP patients according to fracture risk and disease severity, which may help in routine clinical practice to make suitable treatment decisions based on reliable parameters of bone structure and mineralization. Furthermore, we were able to show that non-weight-bearing regions are more appropriate to measure the effect of HPP due to the lower stimulus for bone formation and mineralization by mechanical loading.

## Supplementary Information

Below is the link to the electronic supplementary material.

Supplementary file1 (DOCX 189 KB)
