# Supplementary material for: Radial HR-pQCT and Finite Element Analysis in HPP Patients are Superior in Identifying Susceptibility to Fracture-Associated Skeletal Affections Compared to DXA and Laboratory Tests
Source: Calcif Tissue Int. 2023 May 6;112(6):691–703. doi: 10.1007/s00223-023-01082-3 (PMC10198909; doi:10.1007/s00223-023-01082-3)
Supplement: Supplementary file 1 — Supplementary file1 (DOCX 189 KB) [file 223_2023_1082_MOESM1_ESM.docx]

**Supplemental table 1:** Specifics of each individual included in the analysis after exclusion of motion-artifact-corrupted patients. Unknown information is marked “-“. Dietary specifics are lact. int = lactose intolerance. Mutational class is stated according to ACMG if known. Otherwise, ClinVar I is stated in reference to UniProtVar and stated as “path” if pathogenic and “conf” if information is conflicting.
